# Photoacoustic-Driven Retrieval of Complex Refractive Indices for Absorbing Aerosols

**DOI:** 10.1021/acs.analchem.5c04788

**Published:** 2025-11-26

**Authors:** Abdul Rahman, Muhammad Haseeb Raza, Szabolcs Hodovány, Rana Khawar Ashfaq, Jamshed Saeed Shah, Boglarka Nyerges, Peter Petrik, Cheng Tung Chong, Muhammad Qasim Mehmood, Jichuan Xiong, Gábor Szabó, Zoltan Bozoki, Tibor Ajtai

**Affiliations:** † Department of Optics and Quantum Electronics, 316425 University of Szeged, 9. Dóm Square, Szeged 6720, Hungary; ‡ HUN-REN-SZTE Research Group for Photoacoustic Monitoring of Environmental Processes, 9. Dóm Square, Szeged 6720, Hungary; § Institute of Technical Physics and Materials Science, HUN-REN Centre for Energy Research, Budapest 1121, Hungary; ∥ China-UK Low Carbon College, 12474 Shanghai Jiao Tong University, No. 3, Yinlian Rd, Lingang, Shanghai 201306, China; ⊥ Department of Electrical Engineering, 434888 Information Technology University of Punjab (ITU), Lahore 54000, Pakistan; # School of Electronic and Optical Engineering, 12436 Nanjing University of Science and Technology, 200 Xiaolingwei Street, Nanjing, Jiangsu 210094, China

## Abstract

Accurate determination of the aerosol complex refractive index (RI) is crucial for modeling atmospheric radiative forcing, yet it remains challenging due to limitations in conventional measurement techniques. This study demonstrates a novel and robust methodology for retrieving wavelength-dependent complex RI (*m* = *n* + *ik*) through integrated, simultaneous measurements of aerosol absorption, scattering, and size distribution. We combine multiwavelength photoacoustic spectroscopy (PAS) for filter-free absorption coefficients, integrating nephelometry for angular-resolved scattering, and scanning mobility particle sizing (SMPS) for high-resolution size distribution measurements. Using diethyl-hexyl-sebacate (DEHS) aerosols as a benchmark, we introduce an inverse algorithm based on Rayleigh theory in the near-IR to visible range and Modified Anomalous Diffraction Theory (MADT) in the UV wavelength regime for RI retrieval, overcoming Mie theory instabilities for RI retrieval in the so-called transition region, where particle dimensions are comparable to wavelength. The retrieved RI values agree with bulk ellipsometry measurements within Δ*n* = 0.0011 and Δ*k* = 0.00025. The integrated PAS–nephelometer–SMPS approach establishes a new paradigm for in situ aerosol RI determination with direct applications in climate modeling and remote sensing validation.

## Graphical abstract

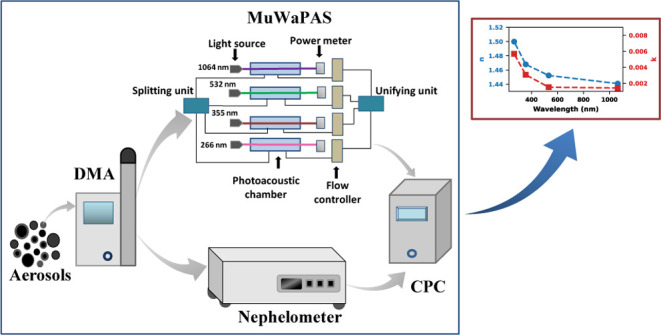

## 1 Introduction

Atmospheric aerosols significantly influence Earth’s radiative balance, air quality, and human health by scattering and absorbing solar radiation.^1,2^ The optical properties of aerosols, such as extinction, scattering, and absorption, as well as complex refractive index (RI), are fundamental parameters for accurately modeling aerosol radiation interactions in climate systems^3^ and for quantitative retrieval of aerosol characteristics from remote sensing observations.^4^ Accurate determination of the optical properties of ambient aerosols is essential for analyzing their impacts on human health and environmental systems.^2^ Consequently, the development of instrumentation capable of precise, real-time in situ measurement of these optical properties is a vital area of research.^5^ However, direct measurement of wavelength-dependent aerosol optical properties remains challenging due to their dependence on dynamically changing parameters like size distribution, morphology, mixing state, and chemical composition.^6^

Current methods for measuring aerosol light extinction, such as long-path cells and cavity-enhanced absorption spectroscopy (CEAS) techniques—including cavity ring-down spectroscopy (CRDS), incoherent broadband CEAS (IBBCEAS), and cavity attenuated phase shift spectroscopy (CAPS)—face significant limitations.^7–10^ While CRDS and CEAS can retrieve refractive indices, they measure only total extinction (scattering plus absorption), failing to distinguish absorbing from nonabsorbing aerosols at specific particle sizes.^11–13^ Although multisize extinction measurements or combined extinction and absorption/scattering data enable RI retrieval, these approaches often lack rigorous validation, particularly for absorbing aerosols with variable imaginary RI components.^14,15^ Moreover, CEAS methods require precise alignment of costly, fragile, and high-finesse cavities and are bulky, limiting their suitability for field applications in harsh or mobile environments. Similarly, filter-based absorption techniques, such as the aethalometer and Multi-Angle Absorption Photometer (MAAP), introduce 20–30% uncertainties due to filter loading and scattering artifacts, underscoring the need for real-time, filter-free solutions.^16–18^ Sequential measurements with optimized instruments can further exacerbate errors, as changing environmental conditions (e.g., temperature, humidity) or inconsistent air sampling alter aerosol properties, leading to ±20–50% uncertainties in radiative forcing due to RI estimation errors (±0.05 in the real part, ±0.01 in the imaginary part).^19–22^ These uncertainties vary with aerosol composition, size, and environment, necessitating the use of integrated, real-time instruments that measure both optical and chemical properties simultaneously to minimize artifacts and enable accurate monitoring under diverse conditions.

Photoacoustic spectroscopy (PAS) has emerged as the preeminent method for accurate, filter-free determination of aerosol light absorption, achieving broad scientific consensus as the most suitable technique for precise measurement of free-floating particles.^23–29^ The PAS technique relies on the photoacoustic effect: when an amplitude-modulated laser beam illuminates particles, they absorb a portion of the incident energy. This absorbed energy is rapidly released as heat to the surrounding gas, generating pressure waves (sound) with an intensity proportional to the modulated laser power. These pressure waves are detected in real time by using a sensitive acoustic sensor. The advent of multiwavelength PAS instruments has further expanded their utility, enabling novel possibilities for qualitative investigations of light-absorbing carbonaceous (LAC) particulate matter and the source apportionment of ambient air.^30–35^ PAS quantifies absorption by directly detecting pressure waves generated from laser-induced heating within the aerosol sample, inherently avoiding biases associated with particle scattering or filter interactions that plague other methods.^36^

Combining PAS for absorption, nephelometry for scattering, and scanning mobility particle sizers (SMPS) for size distribution measurements allows us to calculate the complex refractive index (*n* and *k*) of aerosols.^3,37,38^ This approach is further demonstrated through the determination of the wavelength-dependent refractive index of mineral dust under controlled laboratory conditions, using online instruments and a Mie theory-based algorithm.^39^ However, using Mie theory to retrieve *n* and *k* from these combined measurements is challenging because small errors or assumptions about particle shape and uniformity can lead to unreliable results, making it an ill-posed problem.^11,40,41^ Past studies have used optimization methods or other techniques to improve results, but uncertainties remain.^42–44^ Several studies have employed inverse Mie theory to retrieve the complex RI of aerosols by combining measured optical properties (e.g., extinction, scattering, or absorption coefficients) with particle size distributions.^44–49^ However, Mie theory’s reliance on infinite series expansions makes it computationally expensive and numerically unstable, specifically for particles with sizes comparable to the wavelength (*d* ≈ λ), while also neglecting near-field interactions and edge-diffraction effects. To address these limitations, we propose (i) an inverse Rayleigh–Debye approximation for near-IR to visible wavelengths (*d* ≪ λ) and (ii) a novel inverse algorithm based on modified anomalous diffraction theory (MADT) for UV–visible wavelengths optimized for absorbing aerosols with moderate size parameters (*d* ∼ λ).

We demonstrate a robust, real-time instrumentation framework for retrieving the wavelength-dependent complex RI of aerosols by integrating PAS—extended into the UV—with nephelometry and SMPS, enabling parallel in situ measurements across a broad spectral range. We test this framework using DEHS aerosols, applying inverse Rayleigh theory for larger wavelengths and novel inverse MADT for the UV region to overcome Mie instability at (*d* ∼ λ). Moreover, the retrieved aerosol RI values are cross-validated with DEHS bulk-phase ellipsometry measurements.

## 2 Instrumentation

The integrated experimental setup for simultaneous measurement of aerosol absorption, scattering, size distribution, and dilution is schematically shown in Figure 1. Optical absorption coefficients across four wavelengths were quantified using a custom-built multiwavelength photoacoustic spectrometer (MuWaPAS).^50^ This instrument employs four identical measurement cells operating at 266, 355, 532, and 1064 nm, with each cell maintaining a flow rate of 0.5 L/min. The detection mechanism relies on the photoacoustic effect: when amplitude-modulated laser light is absorbed by particles, the resulting pressure waves are converted to electrical signals via sensitive microphones. These signals are transformed into optical absorption coefficient (OAC) values using experimentally determined calibration factors.^50^ The instrument demonstrates sensitivities (1σ) of <1 Mm^–1^ at 1064 nm, ∼5 Mm^–1^ at 532 nm, ∼23 Mm^–1^ at 355 nm, and 35 Mm^–1^ at 266 nm, corresponding to mass sensitivities ≤1 μg/m^3^, with wavelength-specific measurement uncertainties of 9% (266 nm), 11% (355 nm), 4% (532 nm), and 22% (1064 nm). Detailed performance characteristics are documented elsewhere.^50,51^ Complementing absorption measurements, aerosol scattering coefficients were determined using a TSI model 3563 Integrating Nephelometer, which performs angular integration of scattered light at three defined wavelengths.^52^ Prior to the experiments, the nephelometer was calibrated with CO_2_ gas to establish traceable reference scattering signals and ensure quantification accuracy.

**Figure 1:**
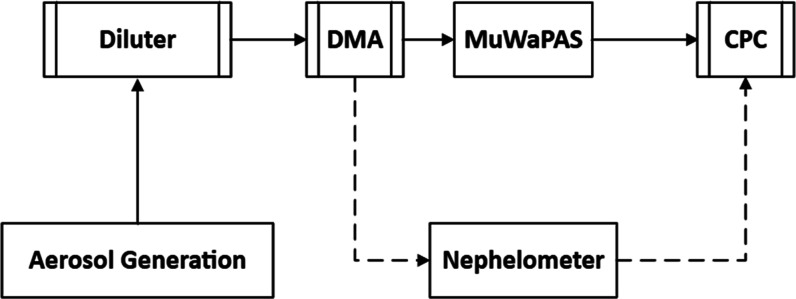
Schematics of the proposed setup. DMA, MuWaPAS, and CPC correspond to the differential mobility analyzer, multiwavelength photoacoustic spectrometer, and condensation particle counter, respectively. The solid lines correspond to absorption efficiency, and the dotted lines correspond to scattering efficiency measurements.

DEHS aerosols were selected as the test medium due to their low vapor pressure and high boiling point, which ensure particle stability and minimize evaporation artifacts during measurements. The TOPAS ATM 220 aerosol generator (TOPAS GmbH, Germany) produced DEHS particles via pneumatic atomization of a pure DEHS solution, with manufacturer-calibrated settings ensuring stable output. Generated aerosols were diluted by a factor of 10 using a PALAS GmbH VKL10 ejector diluter in isokinetic mode, preserving particle representativeness while achieving optimal concentrations for optical characterization. Throughout dispersion, DEHS particles maintained a spherical morphology and neutral charge state to minimize external interactions and ensure consistency in optical behavior. Particle size distributions spanning 11.1 to 1083.3 nm were measured using a Scanning Mobility Particle Sizer (SMPS, TSI 3938). This system consists of a differential mobility analyzer (DMA no. 3082) for electrical mobility-based size classification and a condensation particle counter (CPC no. 3575) for counting size-selected particles. The SMPS achieved resolutions of ±0.5 at 11.1 nm and ±30 at 1083.3 nm. Data integrity was ensured through a real-time coincidence correction to mitigate shielding artifacts and the application of multiple charge compensation algorithms throughout all measurements.

## 3 Results and Discussions

### 3.1 Refractive Index Retrieval Methodology

The determination of aerosol optical properties presents an intricate inverse problem, elegantly analogized to “describing a dragon from its tracks”.^3^ Our methodology solves this inverse problem (retrieving wavelength-dependent RI of absorbing aerosols) through a novel approach that combines simultaneous measurements from an integrated experimental setup of PAS, nephelometry, and SMPS. This unique combination provides the three fundamental inputs required for rigorous refractive index retrieval: (1) direct, wavelength-resolved OAC from PAS, (2) angular-integrated scattering coefficients from nephelometry, and (3) high-resolution size distribution data from SMPS. The inverse algorithm then reconciles these experimental measurements with theoretical predictions to retrieve the complex refractive index. For particles significantly smaller than the wavelength of the incident light, we employ Rayleigh scattering theory, which is particularly relevant for atmospheric aerosols due to their prolonged residence times and prevalence in combustion emissions like black carbon.^1^ The inverse calculation in this regime utilizes the measured OAC from PAS and scattering coefficients from nephelometry combined with the SMPS-derived size distribution to solve for the refractive index components n and k through the closed-form Rayleigh relationships. In this regime, absorption is described as an incoherent process of oscillating dipole damping, which converts electromagnetic energy into thermal energy.^5^ Thus, the single-particle absorption cross-section can be defined as the sum of the individual scatterers’ cross sections, proportional to the particle’s volume. The ensemble-averaged absorption coefficient α_*abs*_ (1/cm) within a resonator (photoacoustic cell) can be expressed as

1 $$\alpha_{abs}=\frac{\sum_{j=1}^{n}\sigma_{{abs}_{j}}}{V}=N\sigma_{abs}$$

where *N* is the number concentration (#/cm^3^), $\sigma_{abs\text{\_}j}$ is the absorption cross-section of the *j*_th_ particle, and *V* is the total volume occupied by the ensemble. The absorption efficiency in the Rayleigh regime, defined as the ratio of absorption cross-section to the geometric cross-section, is given by

2 $$Q_{abs}^{Ray}=\frac{\sigma_{abs}}{\sigma_{geo}}=8\pi \frac{r}{\lambda }Im\left(\frac{m^{2}-1}{m^{2}+2}\right)$$

σ_geo_ is the geometric cross-section of a particle, and for a spherical particle, it is π*r* ^2^. Concurrently, the particle scattering cross-section (σ_sca_) of the particle arises from the coherent superposition of the individual scattering amplitudes $\sqrt{\sigma_{sca\text{\_}j}}$ , with $\sigma_{sca\text{\_}j}$ being the scattering cross-section of the *j*_th_ particle.^5^ The scattering coefficient α_sca_ can be written as

3 $$\alpha_{sca}=\frac{{\left(\sum_{j=1}^{n}\sqrt{\sigma_{{sca}_{j}}}\right)}^{2}}{V}$$

The corresponding scattering efficiency is written as in the following

4 $$Q_{sca}^{Ray}=\frac{\sigma_{sca}}{\sigma_{geometric}}=\frac{128}{3}\pi^{4}\frac{r^{4}}{\lambda^{4}}{\left|\frac{m^{2}-1}{m^{2}+2}\right|}^{2}$$

As particle dimensions approach the wavelength scale, the Rayleigh approximation becomes inadequate due to emerging phase differences between scattered wavefronts and a transition from the volume-dominated absorption of particles to surface-mediated absorption. This transition regime exhibits complex optical behavior featuring interference patterns in extinction spectra, necessitating a more sophisticated theoretical treatment. We adopt modified anomalous diffraction theory (MADT),^53,54^ which extends anomalous diffraction theory (ADT) by incorporating three critical physical phenomena: internal reflections, photon tunneling through near-grazing incidence trajectories, and edge diffraction contributions. The MADT is particularly effective when the imaginary refractive index component *k* is substantially smaller than n-1, as characteristic of weakly absorbing organic aerosols like DEHS.^53^ The MADT formulation expresses dimensionless absorption efficiency as

5 $$Q_{abs}^{MADT}=\left(1+C_{1}+C_{2}\right)Q_{abs}^{ADT}$$

where *Q*_abs_^ADT^ is the absorption efficiency defined by ADT and captures the baseline anomalous diffraction behavior with $\tau =8\pi k\frac{r}{\lambda }$

6 $$Q_{abs}^{ADT}=\left(1+\frac{2}{\tau }e^{-\tau }+\frac{2}{\tau^{2}}\left(e^{-\tau }-1\right)\right)$$

The term *C*_1_ accounts for internal reflections; the term *C*_2_ models photon tunneling effects and is dependent on the particle size and RI.^53,55^

7 $$C_{1}=\frac{1}{4}\left(1+e^{-1167k}\right)\left(1-Q_{abs}^{ADT}\right)$$

8 $$C_{2}=\sqrt{\frac{4\xi r}{\lambda }}\left(e^{0.5-2\xi r/\lambda }\right)\left(0.7393n-0.6069\right),,\xi =\frac{1}{4}+0.61{\left(1-e^{8\pi k/3}\right)}^{2}$$

The scattering counterpart *Q*_sca_^MADT^ incorporates wave optics corrections through a modified extinction term

9 $$Q_{sca}^{MADT}=\left(1+\frac{1}{2}C_{2}\right)Q_{ext}^{ADT}+C_{edge}-Q_{abs}^{MADT}$$

where the *C*_edge_ term contains edge diffraction contributions.^56,57^ The expressions of *C*_edge_ and *Q*_ext_^ADT^ can be written as in the following

10 $$C_{edge}=\left(1-e^{-0.062\pi r/\lambda }\right){\left(\frac{2\pi r}{\lambda }\right)}^{-2/3}$$

11 $$Q_{ext}^{ADT}=2-4\frac{e^{-\rho \tan\beta }\cos\beta }{\rho }\;\sin\left(\rho -\beta \right)+4\frac{e^{-\rho \tan\beta }\cos^{2}\beta }{\rho^{2}}\;\cos\left(\rho -2\beta \right)+4\frac{\cos^{2}\beta }{\rho^{2}}\;\cos2\beta$$

The ρ is a phase-shift parameter,^58^ defined as $\rho =\frac{4\pi r}{\lambda }\left(n-1\right)$ and $\beta =\arctan\left(\frac{k}{n-1}\right)$ . The complex refractive index components (*n*, *k*) were retrieved through constrained optimization using the Limited-memory Broyden–Fletcher–Goldfarb–Shanno Bound (L-BFGS-B) algorithm.^59^ This gradient-based approach minimizes the residual error between measured and theoretically predicted optical properties while respecting physical bounds on n and k. The implementation proceeded as follows

12 $$error={\left(\frac{\alpha_{abs,cal}-\alpha_{abs,measured}}{\alpha_{abs,measured}}\right)}^{2}+{\left(\frac{\alpha_{sca,cal}-\alpha_{sca,measured}}{\alpha_{sca,measured}}\right)}^{2}$$

where α_abs,cal_ and α_sca,cal_ are computed using Rayleigh theory and MADT for given (*n*, *k*). The methodology’s robustness stems from its physical basis in wave optics rather than empirical fitting, enabling reliable extrapolation across the atmospherically critical 200–1000 nm spectral range. Critically, two distinct approaches were employed: the well-established Rayleigh theory for IR/visible wavelengths (1064 and 532 nm) and the MADT for UV wavelengths (355 and 266 nm). This wavelength-specific strategy optimizes accuracy by addressing the following theoretical limitations: Mie theory assumes homogeneous spheres but becomes computationally unstable for medium-sized parameters, whereas MADT accounts for wave optic effects that are dominant in the UV regime.

### 3.2 Aerosol Size Distribution and Stability

Accurate characterization of aerosol size distributions is critical for optical property measurements, as both absorption and scattering efficiencies exhibit strong size dependence.^3,60^ To characterize aerosol properties, the SMPS is commonly used to measure the mobility size distribution data of both environmental and laboratory-generated aerosols.^33–35,61^ For RI retrieval, size-resolved analysis was performed using a DMA coupled with a CPC. The DMA was calibrated at +180 V for 107 nm particles and +496 V for 200 nm particles—voltages determined from the equilibrium charge distribution and the mobility diameter relationship.^62^ Particles were passed through the integrated PAS–nephelometer system for optical characterization, while downstream CPC measurements confirmed good stability. Specifically, 107 nm aerosols maintained a concentration of 51,250 ± 3000 cm^–3^ with a 5.8% coefficient of variation over 2 h. A monomodal size distribution with a full width at half-maximum (fwhm) of 3.6 nm was achieved. As shown in Figure 2, temporal concentration profiles demonstrated minimal fluctuation (<5% CV). This consistency was achieved through isokinetic dilution and thermodynamic control. The integrated approach validates our size-resolved methodology for complex refractive index determination, with stability metrics confirming that observed optical properties reflect intrinsic particle characteristics rather than system artifacts.

**Figure 2:**
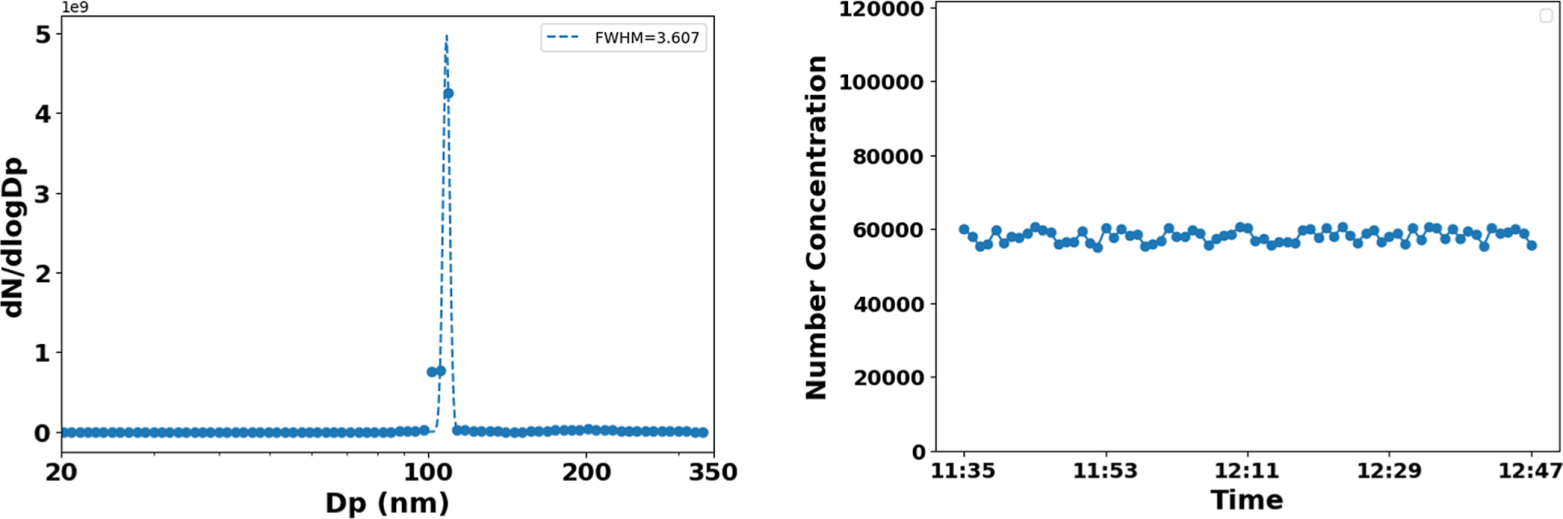
Size selection of the particles from DMA (107 nm) (left). Temporal stability of the number concentration measurements using CPC (right).

### 3.3 Optical Measurements and Retrieval of Complex RI

Aerosol absorption coefficients were quantified using a custom four-wavelength photoacoustic spectrometer based on the differential dual-resonator design.^36^ The system employs a Q-switched Nd:YAG laser (1064 nm fundamental wavelength) with second, third, and fourth harmonic generation via lithium triborate (LBO) and beta barium borate (BBO) nonlinear crystals to produce output at 532, 355, and 266 nm. Each wavelength was directed to dedicated measurement PA cells featuring antireflection-coated fused silica windows, minimizing reflection losses (<0.2% per surface). All four identical cells contain matched cylindrical resonators (110 mm length × 5 mm diameter), a differential microphone configuration for coherent noise rejection, and integrated buffer volumes that damp PA noises. The PA cells calibration followed established protocols using NO_2_ gas absorption at 532 nm.^24,50^ The sample flow through each cell was maintained at 0.5 L/min via isokinetic sampling, ensuring representative aerosol delivery without size segregation. Signal processing involved dual-phase lock-in amplification at resonance frequencies (∼4 kHz) and Fourier transformation of differential microphone signals. Although PAS is recognized as one of the most reliable techniques for measuring the absorption of in situ aerosols with minimal uncertainty, several factors still contribute to the overall measurement uncertainty. The main sources of uncertainty arise from (i) uncertainties in the mass concentration within the resonator, i.e., the monomodal size distribution of DEHS aerosols measured using a differential mobility analyzer (DMA), and (ii) instrumental noise originating from the resonator walls and window absorption. The relative uncertainties estimated from DEHS aerosol measurements are 1.40% at 1064 nm, 1.14% at 532 nm, 1.49% at 355 nm, and 1.68% at 266 nm. Figure 3 presents the measured OAC for DEHS aerosols across all wavelengths. The observed absorption dependence shows a characteristic increase toward shorter wavelengths (Figure 3).

**Figure 3:**
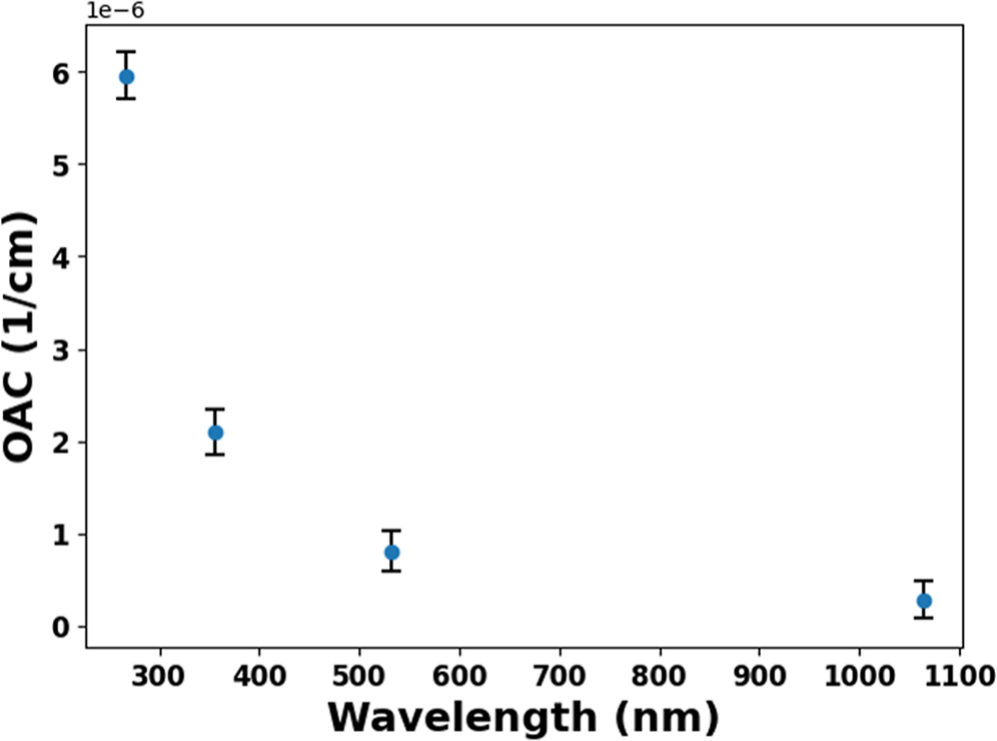
Wavelength-dependent optical absorption coefficients of DEHS aerosols.

The complementary scattering coefficient measurements were performed using an integrating nephelometer operating at 450, 550, and 635 nm. This instrument employs orthogonal illumination-detection geometry where a temperature-stabilized halogen lamp (3000 K color temperature) illuminates the sample volume, with three photomultiplier tubes detecting scattered light across angular sectors: 7–90° (forward scattering), 90–173° (backward scattering), and 7–173° (total scattering). For 107 nm DEHS aerosols, scattering coefficients measured by nephelometry at 450, 550, and 635 nm were empirically extrapolated to PA spectroscopy wavelengths (1064, 532, 355, and 266 nm) based on observed wavelength-dependent scattering trends. Figure 4 shows the complete scattering spectrum, revealing strong wavelength dependence toward UV wavelengths.

**Figure 4:**
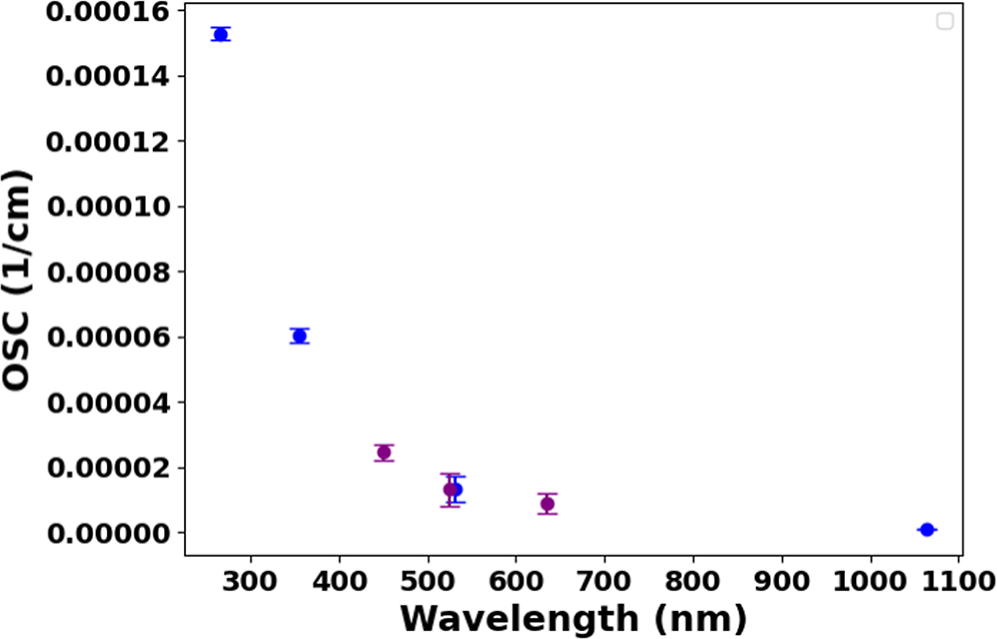
Spectral scattering coefficients of DEHS aerosols. The blue data points indicate extrapolated values.

This comprehensive approach provides synchronized, wavelength-resolved absorption and scattering coefficients across 266–1064 nm, enabling robust refractive index retrieval while eliminating temporal artifacts through <1s measurement synchronization as discussed in Section 3.1.

### 3.4 Validation against Bulk Ellipsometry

To validate our proposed real-time method, we performed comparative bulk-phase measurements using spectroscopic ellipsometry. Unlike nanoparticle suspensions, where absorption and scattering exhibit strong shape dependence,^3^ bulk-phase liquids provide unambiguous optical properties, enabling direct validation. The optical properties of DEHS were characterized on an open liquid surface using a J.A. Woollam M2000DI rotating compensator spectroscopic ellipsometer. Given the inherent challenge of tilting a free liquid surface, instrument-level tilt adjustments were implemented to achieve precise angular alignment.^63^ Backside reflections were mitigated by employing a liquid layer thickness (>3 mm) exceeding the incident beam diameter. Measurements at incidence angles of 60°, 65°, and 70° enhanced data robustness and reduced uncertainty. For direct comparison with our method, the real and imaginary parts of the refractive index were fitted using the b-spline model,^64^ forcing Kramers–Kronig consistency that uses polynomial nodes spaced at 0.3 eV intervals. This ensured physical fidelity in the dispersion analysis. Sensitivity for both refractive index components exceeded 0.001 across visible and near-IR wavelengths, decreasing to 0.01 in the UV regime. The excellent agreement between our absorption and scattering values obtained from the proposed online method and ellipsometry results is demonstrated in Figure 5.

**Figure 5:**
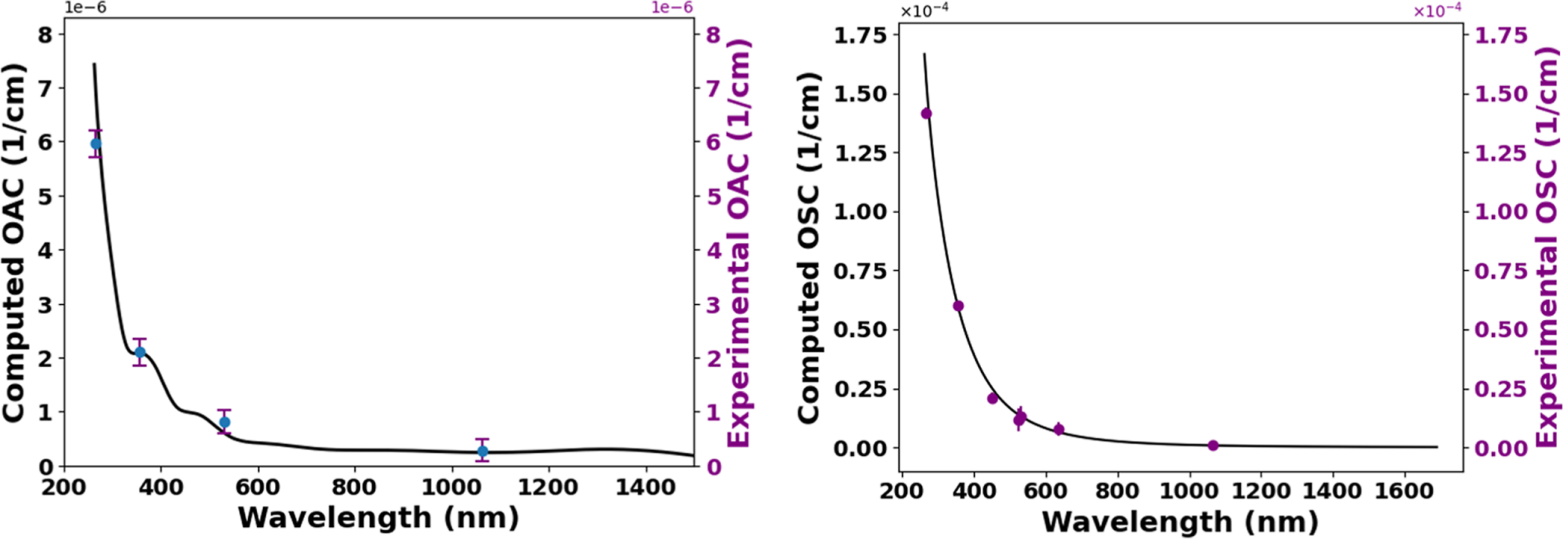
Comparison of the complex RI values obtained using ellipsometry and the experimentally evaluated *n* and *k* values from the proposed integrated infrastructure.

The retrieved complex RI values from the proposed integrated online technique and bulk-phase measurement are listed in Table 1. The comparison of complex RI values validates the proposed methodology against bulk-phase ellipsometry measurements for DEHS aerosols (Table 1). The table lists the real and imaginary components of the RI obtained from ellipsometry, alongside uncertainties Δ*n* and Δ*k* at four wavelengths (1064, 532, 355, and 266 nm). These are compared with RI values retrieved using the integrated PAS-nephelometry-SMPS system, employing inverse Rayleigh theory for near-IR/visible wavelengths and MADT for the UV regime. The excellent agreement between the proposed methodology and bulk-phase measurements, with deviations of Δ*n* ≤ 0.0011 and Δ*k* ≤ 0.00025, confirms the robustness and accuracy of the approach across the 266–1064 nm spectral range.

**Table 1:** The Retrieved *n* and *k* Values from the Aerosol Phase Using the Proposed Photoacoustic-Driven Methodology and from the Bulk Phase Using Ellipsometry Measurements, along with the Uncertainty between the Two Methodologies

	aerosol phase results		bulk phase results		uncertainty	
wavelength (nm)	*n*	*k*	*n*	*k*	Δ*n*	Δ ***k***
1064	1.440855	0.001414	1.439742	0.001420	1.1 × 10^–3^	6.1 × 10^–6^
532	1.452273	0.001513	1.452107	0.001492	1.65 × 10^–4^	2.5 × 10^–5^
355	1.467895	0.003074	1.467753	0.002980	1.4 × 10^–4^	9.4 × 10^–5^
266	1.499903	0.005687	1.499935	0.005966	3.2 × 10^–5^	2.79 × 10^–5^

## 4 Conclusion

This study demonstrates a robust, integrated methodology for retrieving wavelength-dependent complex refractive indices of absorbing aerosols through simultaneous measurements using PAS, nephelometry, and SMPS instruments. The customized multiwavelength PAS infrastructure ensures precise absorption measurements extended into the deep UV spectrum (266–1064 nm). The real-time, filter-free PAS–nephelometer–SMPS system facilitates the dynamic monitoring of aerosol optical properties under varying environmental conditions, including humidity, temperature, and chemical composition. By employing inverse Rayleigh theory for near-infrared and visible wavelengths (1064 and 532 nm) and a novel MADT algorithm for the UV regime (355 and 266 nm), this methodology addresses numerical instabilities inherent in Mie-based retrievals in the transition region where particle sizes approximate the wavelength (*d* ≈ λ). This approach provides physically meaningful absorption coefficients in this critical region. Validation against bulk-phase ellipsometry for DEHS aerosols demonstrates exceptional accuracy, with deviations of Δ*n* ≤ 0.0011 and Δ*k* ≤ 0.00025 across the 266 to 1064 nm range, confirming the reliability of the retrieved RIs and overcoming challenges associated with RI retrieval from bulk and aerosol phase comparisons.

The field-deployable design of this methodology enhances its applicability for validating satellite-based aerosol retrievals, thereby improving the accuracy of remote sensing data critical for climate modeling and air quality assessments. By delivering precise RI values, this approach significantly reduces uncertainties in radiative forcing estimates, which currently suffer from errors of ±20–50% due to RI inaccuracies. This methodology addresses critical gaps in aerosol radiative forcing models and enables robust validation of remote sensing data, paving the way for enhanced climate predictions and air quality monitoring. Its potential for further development, particularly in characterizing complex aerosol morphologies, positions it as a valuable tool for advancing atmospheric science research.
